# Key connection between gravitational instability in physical gels and granular media

**DOI:** 10.1038/s41598-022-10045-x

**Published:** 2022-04-15

**Authors:** Kazuya U. Kobayashi, Rei Kurita

**Affiliations:** 1 Department of Mechanical Systems Engineering, Tokyo University of Agriculture and Technology, 2-24-16 Nakacho, Koganei-city, Tokyo Japan; 2 Department of Physics, Tokyo Metropolitan University, 1-1 Minami-Oosawa, Hachioji-city, Tokyo Japan

**Keywords:** Fluid dynamics, Statistical physics, thermodynamics and nonlinear dynamics

## Abstract

We study gravitationally-driven (Rayleigh–Taylor-like) instability in physical gels as a model for the behavior of granular media falling under gravity; physical gels have a structural elasticity and may be fluidized, capturing both the solid and liquid properties of granular systems. Though ubiquitous in both industrial and natural contexts, the unique static and dynamic properties of granular media remain poorly understood. Under the action of a gravitational force, granular materials may flow while exhibiting heterogeneous rigidity, as seen during e.g., avalanches or landslides. Though the onset of this gravitational “instability” has been addressed, the mechanism behind its incidence is not yet understood. We find key quantitative similarities between Rayleigh–Taylor-like instability in physical gels and granular systems. In particular, we identify a common scaling law, showing that the instability is chiefly governed by the thickness of the flowable region.

## Introduction

A granular medium is a closely packed collection of a large number of grains, often tens of microns to a few millimeters in size. They are ubiquitous in industrial products and processes e.g., pharmaceuticals, paints, cosmetics etc.^1–5^, and also govern a wide range of natural phenomena, including the occurrence of natural disasters such as avalanches, landslides, and soil liquefaction during earthquakes^6,7^. Although granular materials should be described via classical mechanics of many bodies, its statical properties have been unpredictable. The properties are complex due to coupling among a friction between grains, size dispersion, and shape of grains. For example, it is known that external forces propagate via a localized path known as a force chain or stress network; the physical response of granular media may vary not only due to static structural properties but on the history of a procedure^8,9^.

In this article, we focus on the dynamical properties of granular media, in particular the instability of granular systems under gravity. The effect of gravity on granular packings is related with a vast range of natural phenomena such as avalanches^10^, response to impact^11,12^, turbulence^13^, mixing^14^, magma flow, liquefaction, dust explosions and tectonics^6,7^. Both experiments and numerical simulations have established that gravitational instability in granular systems is unique and clearly distinguished from fluid systems^3,4,15–17^. We note three: (i) during the incidence of gravitational instabilities, stress networks are disconnected by the weight of the grains from the vicinity of the grain-gas interface, leading to the formation of a fluid-like layer near the interface. Meanwhile, regions far from the interface stay solid-like due to percolated stress networks. The fluid-like region is then free to fall under gravity^3,4,15–17^; (ii) numerical simulations have shown that the instabilities at the interface for granular systems exhibit short wavelength fluctuations at the beginning and then the wavelength remains short for a long time^17^. For comparison, in fluid systems, the wavelength of the fluctuation is constant at the very beginning, while it subsequently grows by several times over time; (iii) a fingering pattern can be seen, much like a Rayleigh–Taylor instability in fluids. However, in granular materials, new fingers are born between existing fingers^17^. This is never observed in fluid systems^6,18–23^. Despite recent developments in granular physics, the mechanism behind (ii) and (iii) above remains unclear.

Recently, similarities have been identified between phenomena in granular materials and other systems^1,3,4^. For example, it was found that jamming in granular systems may be related to the glass transition^24,25^. Analogies between the temperature and the packing of the granular media has been discussed^26–28^. In addition, granular flow has also been compared with the flow of complex fluids^29,30^ and instability phenomena in fluids^31–34^. Such analogues are expected to provide key physical insights. Here, we studied the behavior of physical gels and compare it to the behavior of granular systems. In gels, polymer chains are percolated below the sol-gel temperature $T_c$; by heating the gel above $T_c$, the system can be readily fluidized. Note the similarities with granular media; the percolation of polymer chains is similar to the connectivity of the stress network between grains, while fluidization above $T_c$ may be framed as a close analogue to fluidization in a packed granular media. Here, we show that there is indeed a close relationship between Rayleigh–Taylor-like instability in physical gels and granular materials. More specifically, our experiments identified a common scaling law, suggesting that Rayleigh–Taylor-like instability in both systems are governed by the thickness of the flowable region.

**Figure 1:**
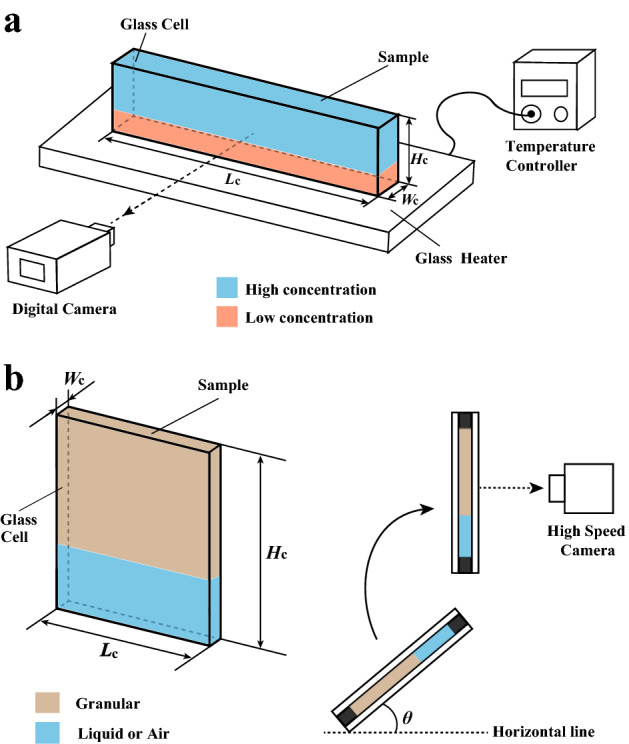
(**a**) Schematic of our experimental setup for gels. A glass sample chamber we use has internal dimensions (H, L, W) = (30 mm, 126 mm, 2.4 mm). Polystyrene latex microspheres are introduced into the upper layer to visualize the flow. Images were recorded with a digital camera. We control the bottom temperature $T_b$ by the heater. (**b**) Schematic of our experimental setup for granular systems. We put grains in the sample chamber (H, L, W) = (150 mm, 75 mm, 1.2 mm) or (90 mm, 65 mm, 2.4 mm) with sedimentation angle $\theta$ and then it is rotated manually.

## Results

### Brief strategy of our experiment for the Rayleigh–Taylor-like instability

**Figure 2:**
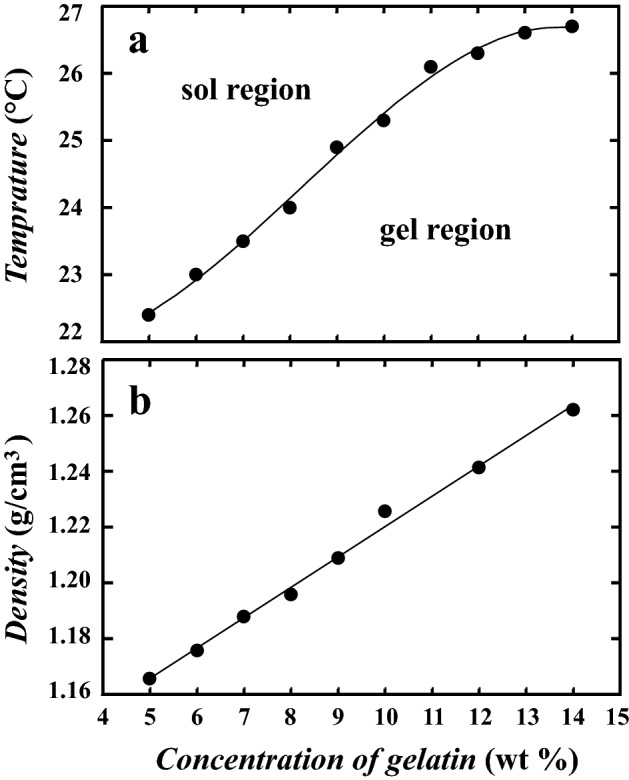
(**a**) Dependence of the sol-gel transition temperature on concentration. (**b**) Dependence of the density of the gelatin solution on concentration. We evaluated the sol-gel transition temperature using a tube inversion method.

Firstly, we introduce brief strategy of our experiment for the Rayleigh–Taylor-like instability. Details of our experimental methods are described in the Materials and Methods section. Our experimental set up for the gel is roughly sketched in Fig. 1a. The thickness of the cell $W_c$ is comparable or smaller than the most unstable wavelength of the Rayleigh–Taylor-like instability $\lambda$. Thus, our sample chamber can be regarded as a quasi two-dimensional Hele-Shaw cell for accurate analysis of flow and structure. The physical gel we studied was a double-layered (upper/lower) structure contained in a microscopy. The lower layer consisted of an aqueous or low concentration solution of gelatin; the upper layer was a high concentration solution. By heating the sample from the bottom surface, the bottom layer is fluidized first and then the upper layer is fluidized from the interface. This led to the gradual incidence of Rayleigh–Taylor-like instability near the interface, while the whole upper layer remained elastic. Note the similarities with gravitational instabilities in granular media. It is worth noting that the interfacial energy between the two gelatin solutions is negligible, much like the interface between air and grains. Our experimental setup makes it possible to observe the formation of the Rayleigh–Taylor-like instability from the very beginning, allowing accurate observation of how the distance between fingers changes over time^23^. In addition, we may also systematically investigate how the gravitational instability changes in response to different viscosity and density gradients by changing the bottom temperature and the composition of the upper and lower layers (see Fig. 2).

In order to observe gravitational instability in granular materials, we used grains which the size is $0.425 \pm 0.150$ mm. Following a previous method reported in Ref.^17^, we homogeneously injected grains at ambient pressure to form a random loose packing in the sample chamber and then this is rotated manually to initiate the instability phenomenon (See Fig. 1b). We also investigate the gravitational instability in granular-water systems. We injected the grains with changing sedimentation angle ($\theta$ = 30°, 45°, 60°, and 90°) in order to roughly control the packing fraction of the random loose packing. The packing fraction is estimated as 0.46 for $\theta$ = 30°, 0.49 for $\theta$ = 45°, 0.54 for $\theta$ = 60°, and 0.56 for $\theta$ = 90°. We note that the packing faction of the random close packing of this grains is 0.58^35^.

**Figure 3:**
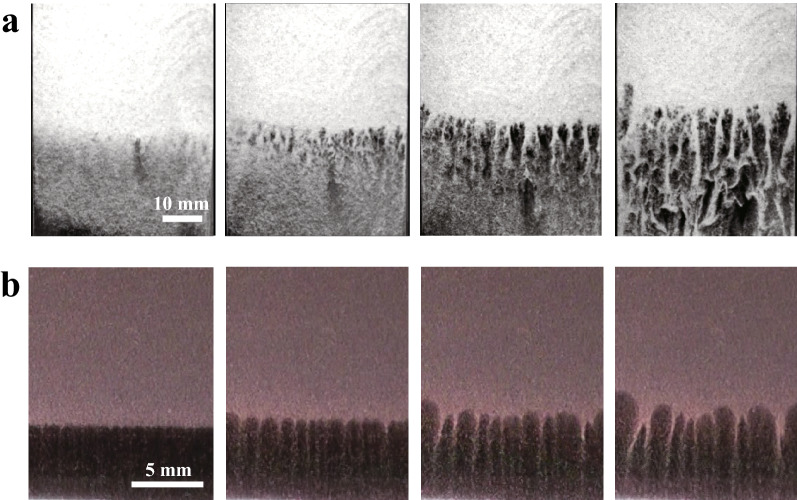
(**a**) Time evolution of the gravitational instability in the granular system. From left to right, *t* = 0.06 s, 0.1 s, 0.14 s, and 0.24 s. (**b**) Time evolution of the gravitational instability in a physical gel. From left to right, *t* = 250 s, 350 s, 425 s, and 450 s. The concentration of the gelatin solution is 6 wt% for the upper layer and 5 wt% for the bottom layer. The base temperature is set to 26 °C. The fingering pattern and growth process in the granular system is qualitatively similar to that in the physical gel system.

### Rayleigh–Taylor-like instability in both gel and granular systems

We begin by showing how for the Rayleigh–Taylor-like instability evolve in both systems. Figure 3a shows the grain-gas system, where the granular layer and air layer are represented in white and black, respectively. *t* = 0 is the moment the sample cell is completely rotated; the whole process takes about 0.3 s. At the beginning ($t<$ 0.06 s), it is observed that particles fall away individually from the grain-air interface. Subsequently, a fingering pattern emerges (*t* = 0.1–0.14 s) and coarsens (*t* = 0.24 s). Note that a new finger is often created between existing fingers during the coarsening process. This seems to be a typical feature of Rayleigh–Taylor-like instability in granular systems. This pattern evolution is consistent with previous results^17^.

**Figure 4:**
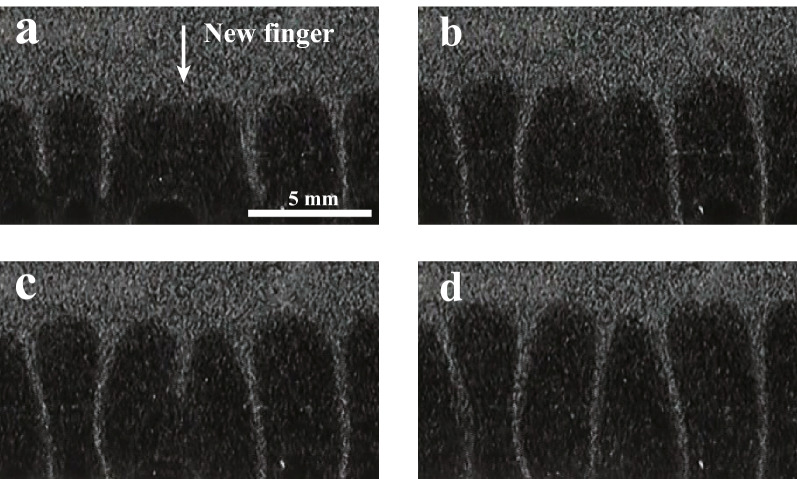
A new finger is intermittently formed during the intermediate stage of the growth of Rayleigh–Taylor-like instability in the gel system. *t* = (**a**) 440 s, (**b**) 460 s, (**c**) 480 s, and (**d**) 510 s. A new finger is often formed when the distance between existing fingers is large. The emergence of the new finger is also observed during the drop of the granular system. We use 6 wt% for the upper layer and 5 wt% for the bottom layer. $T_b$ = 26 °C.

We compare this to Rayleigh–Taylor-like instability in our physical gel system. This is shown in Fig. 3b, where we prepare a 6 wt% upper layer and 5 wt% bottom layer. We set the temperature of the sample base $T_{b}$ to 26 °C at *t* = 0 s. $T_{b}$ is higher than the sol-gel transition temperature $T_c$ (see Fig. 2). At the beginning, sedimentation with a short wavelength fluctuation is observed ($t<$ 250 s). This is followed by the emergence of a fingering pattern (*t* = 350–425 s). Then, these fingers go on to coalesce and coarsen over time (*t* = 450 s). Here, we observe that new fingers form between existing fingers as shown in Fig. 4. All typical features of the gel thus closely resemble those of the granular system, rather than Rayleigh–Taylor instability in fluids.

### The dynamics of the Rayleigh–Taylor-like instability in the gel systems

**Figure 5:**
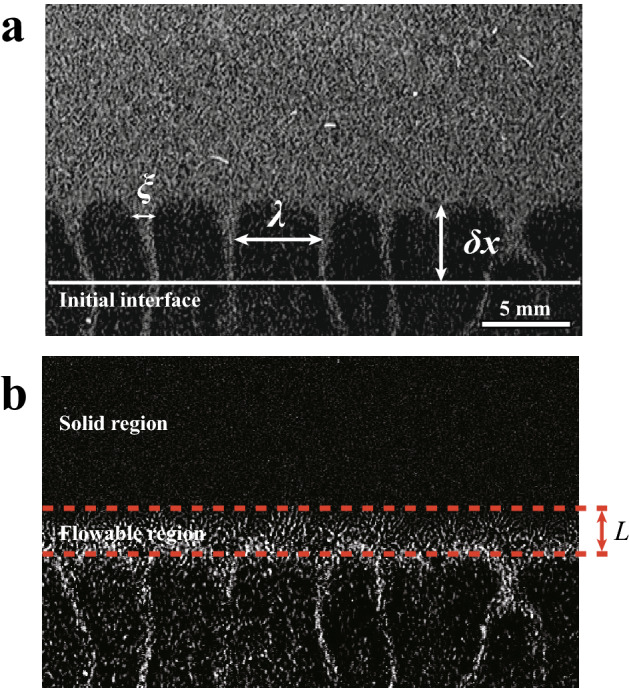
Characteristic parameters for both systems. (**a**) The distance moved by the interface from an initial position, $\delta x$; thickness of the region which can flow, *L*; the mean distance between the fingers, $\lambda$; width of the fingers, $\xi$. The number of the fingers is about 40 at the beginning and it is more than 10 at the end of the experiment. The standard deviation of $\lambda$ is at most 10%. (**b**) The difference between two consecutive images. *L* is thickness of the region which can flow. Solid region is located above the flowable region. Near the interface, the gravitational flow is dominant, but the horizontal flow is negligible.

We proceed to describe the systems quantitatively by extracting some characteristic parameters. We define the distance that the instability interface has moved from its initial position $\delta x$, the thickness of the fluidized region *L*, the mean distance between the fingers $\lambda$, and the width of the fingers $\xi$. These parameters are illustrated in Fig. 5. At the lower boundary of the flowable region, the intensity sharply increases from the bottom. The position of the lower boundary is determined by the center of the interface. The number of the fingers is about 40 at the beginning and it is more than 10 at the end of the experiment. In general, the real space analysis is better than Fourier transform analysis when the number is not large. Thus, $\lambda$ is obtained by averaging the distance between the nearest fingers and the standard deviation of $\lambda$ is at most 10 %. We use the image differencing technique (IDT), which the flow is evaluated by subtracting two consecutive images taken from an image sequence. We determine the higher boundary of the flowable region by IDT (see Fig. 5b), where the region below the resolution of the IDT (0.012 mm/s) is considered as solid state. We note here that the position of the higher boundary is not sensitive to the resolution of the IDT since the velocity drastically decreases at the higher boundary. Then *L* is the distance from the lower boundary of the flowable region to the higher boundary. The main error in $\delta x$ and *L* comes from the width of the interface and it is less than 0.3 mm.

**Figure 6:**
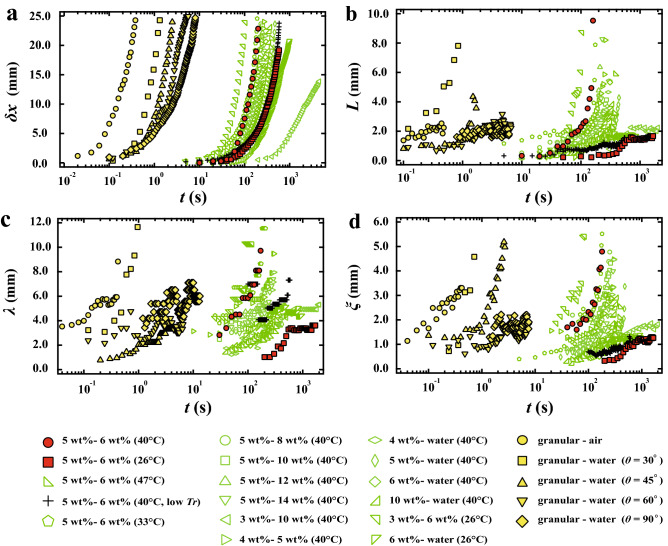
Time evolutions of the gravitational instability for gels and granular systems. (**a**) the front position $\delta x$, (**b**) the thickness of flowable region *L*, (**c**) the mean distance between the fingers $\lambda$, (**d**) the mean width of fingers $\xi$.

We investigate how these parameters evolve over time in different combinations of upper and lower layers and several $T_b$. Figure 6 shows the time evolution of $\delta x$, *L*, $\lambda$, and $\xi$ for gels and granular systems. Looking at the time evolution of $\delta x$ for different combinations of upper and lower layers, we found that the characteristic time over which $\delta x$ varies tends to be larger for higher concentrations of the upper layer. It seems that $\lambda$ and $\xi$ largely scatter. $\lambda$ and $\xi$ change when the fingers collapses or and the new finger emerges between the fingers. Thus, it is not due to the errors of the measurement.

**Figure 7:**
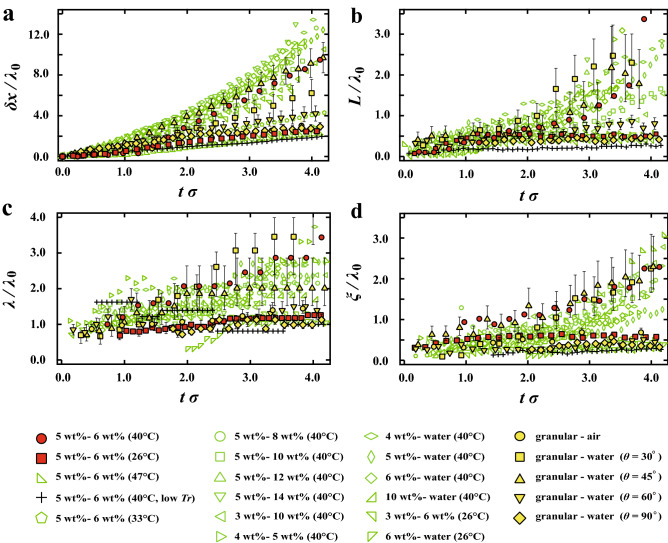
Time evolutions of the instability with respect to normalized time $t\sigma$ in the physical gels and the granular systems. (**a**) normalized front position $\delta x/\lambda _0$, (**b**) normalized thickness of flowable region $L/\lambda _0$, (**c**) normalized mean distance between the fingers $\lambda /\lambda _0$, (**d**) normalized mean width of the fingers $\xi /\lambda _0$. The error bars for the granular systems correspond to the standard deviation of the results.

To reduce the viscosity effect, we normalize the length scale and the time scale of the dynamics. We define $\lambda _0$ and $\sigma$ as the mean distance between the fingers and a growth rate of the fingers at the beginning, respectively. Figure 7a–d show the relationships between each normalized parameter and $t \sigma$. It is found that the curves are not collapsed even if the combinations of upper and lower layers are same. For example, $\delta x/\lambda _0$ in 5–6wt% at $T_b$ = 40 °C increases much faster than that at $T_b$ = 26 °C although the time scale of the dynamics is normalized. It is also found that both $L/\lambda _0$ and $\lambda /\lambda _0$ increase faster at $T_b$ = 40 °C, while they remain almost constant at $T_b$ = 26 °C. It suggests that $T_b$ is a crucial parameter for the Rayleigh–Taylor-like instability in the gel systems. In addition, it is worth noting that there are strong relations among $\delta x / \lambda _0$, $L/\lambda _0$, and $\lambda /\lambda _0$.

### The dynamics of the Rayleigh–Taylor-like instability in the granular systems

Then we investigate the Rayleigh–Taylor-like instability in the granular-air system the granular-water system. The system is rotated manually, suggesting a strong possibility that human error may affect results. Thus, we performed the same experiment with 5 times and confirmed that the results are reproducible. The time evolutions of the Rayleigh–Taylor-like instability in the granular systems are shown as yellow circle symbols in Fig. 6 and Fig. 7. The error bars in Fig. 7a–d correspond to the standard deviation of the results. We also confirm that those dynamics are independent of the thickness of the sample chamber at least in the range of the thickness from 1.2 mm to 4.8 mm. It is found that those evolutions of all the parameters in the granular systems (filled yellow symbols in Fig. 7a–d) are similar to those of the physical gels, although the characteristic time is much faster than that in the gel systems.

### Scaling by the width of the flowable region

**Figure 8:**
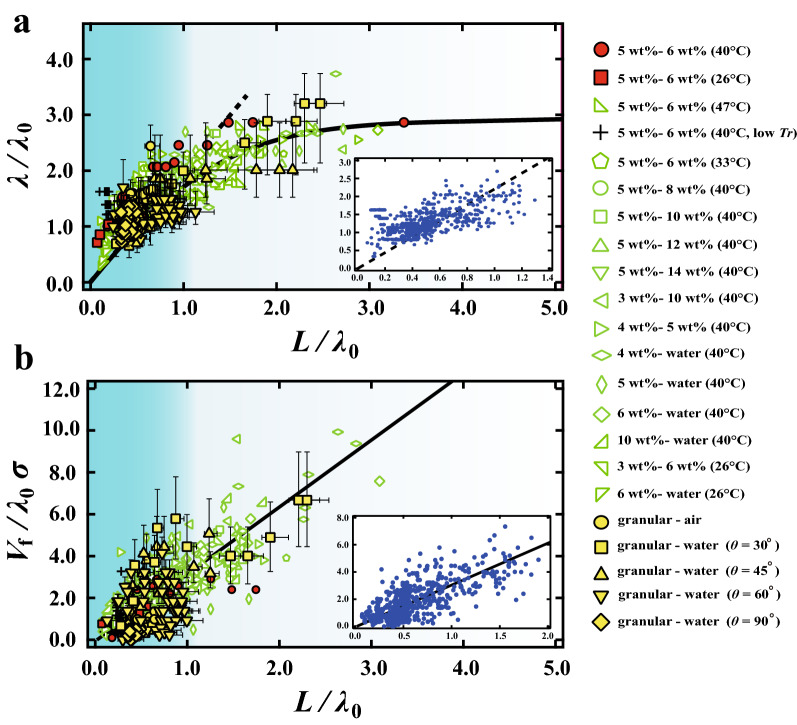
(**a**) $\lambda /\lambda _0$ as a function of $L/\lambda _0$. For smaller $L/\lambda _0$, $\lambda /\lambda _0$ is linear with respect to $L/\lambda _0$ (dashed line in (**a**)). The solid line is an eye guide for the entire process of the Rayleigh–Taylor-like instability. The region colored by blue background corresponds to the linear regime of the Rayleigh–Taylor-like instability. The inset shows $\lambda /\lambda _0$ near the origin. The dots are used in order to see all data clearly. (**b**) $V_f / \lambda _0 \sigma$ as a function of $L/\lambda _0$. The solid line is linear fitting for $V_f / \lambda _0 \sigma$. The inset shows $V_f / \lambda _0 \sigma$ near the origin. The dots are used in order to see all data clearly. $\lambda /\lambda _0$ and $V_f / \lambda _0 \sigma$ in physical gels are scaled by *L*, regardless of $T_b$. Furthermore, $\lambda /\lambda _0$ and $V_f / \lambda _0 \sigma$ in the granular systems are also scaled.

Although $T_b$ is an obvious experimental parameter for the gel, we note that temperature is a negligible parameter for the granular systems. $T_b$ is thus not expected to be the dominant parameter governing the common dynamics seen above. In fluid systems, it has been shown that $\lambda$ can be estimated as $\lambda \sim \epsilon ^{-1/5} H$, where $\epsilon$ and *H* are the viscosity ratio between the upper and lower fluids and the thickness of the thinner fluid, respectively^36^. Note that *H* and *L* are equivalent except for the fact that *L* in our experiment changes over time. Since the viscosity dependence is quite weak ($\sim \epsilon ^{-1/5}$), *L* in our experiment may be a key parameter governing the dynamics of the gravitational instability. Figure 8a shows $\lambda /\lambda _0$ as a function of $L/\lambda _0$ and the inset shows $\lambda /\lambda _0$ near the origin. It is found that $\lambda /\lambda _0$ is almost proportional to $L/\lambda _0$ for smaller $L/\lambda _0$ (blue background region in Fig. 8) regardless of the bottom temperature. We determine the linear regime where the macroscopic convective flow can be neglected (also see “Discussion”). It is difficult to estimate the effect of the macroscopic flow near the limit of the linear regime, thus the limit of the boundary is vague. Here we fitted the data in the linear regime by a liner function and then is obtained that $\lambda = 2.2L$. It is also found that Pearson coefficient is 0.68. The value of $R^2$ is 0.86 when we fixed y-intercept to the origin and it is 0.45 for the linear fitting. Those results suggest that there is a positive correlation between $\lambda$ and *L* although the data is distributed. The result is roughly consistent with a previous experimental result in a low viscosity fluid system, when $\lambda = 1.0 L$ at the beginning^23^.

Furthermore, we define a dynamical parameter $V_f$ as a front velocity of $\delta x$. To normalize the length scale and the time scale of the dynamics, we also define a normalized front velocity $V_f / \lambda _0 \sigma$. Figure 8b shows $V_f / \lambda _0 \sigma$ as a function of $L/\lambda _0$ . We fitted the data by a liner function and then it is obtained that $\lambda = 3.0L$. It is also obtained that the Pearson coefficient is 0.82. The value of $R^2$ is 0.84 when we fixed y-intercept to the origin and it is 0.67 for the linear fitting. Thus, $V_f / \lambda _0 \sigma$ has a positive correlation with $L/\lambda _0$ regardless of various conditions. We note here that the growth rate of the Rayleigh–Taylor-like instability in fluids is proportional to *L*^36^. Consequently, the Rayleigh–Taylor-like instability in the gelatin systems at the linear regime can be understood as a fluid system where the layer thickness changes over time. Importantly, the dynamics in the granular system is almost scaled by *L*. This means that the dynamics of granular sedimentation is also equivalent to a fluid system where the layer thickness changes with time. We will discuss the behaviors after the linear regime and the distribution of the data in the “Discussion” section.

## Discussion

Firstly, we discuss the determining factors for *L* in the gel and the granular systems. The fluidized region in the gelatin system is determined by the location of the isothermal line corresponding to the sol-gel transition temperature. Thus, *L* is determined by thermal conduction i.e., the base temperature $T_b$. This is why the dynamics of the gel system seems to be determined by $T_b$ (see Fig. 6). Meanwhile, *L* in the granular systems seems to be related to the “strength” of the stress network. According to previous works, the structure of the granular system may be converted into the effective temperature (Edwards temperature) $T_e$^26^. When $T_e$ is lower temperature, it means the packing fraction is larger or the structure is close to the random close packing. That is, $T_e$ is lower when the stress network is strong. Here we reconsider our results for the granular system using $T_e$. The effective gravitational force for packing is weak if the sedimentation angle $\theta$ is small and then $T_e$ is higher (the stress network is weak). In fact, it is found that *L* increases much faster when the sedimentation angle is small ($\theta$ = 30° and 45°). This is consistent with a result reported in a previous work, where *L* changes linearly with time in glycerol^15^. In the previous case, $T_e$ may be higher since the sedimentation is quite slow and the packing fraction should be smaller. In addition, we found that the dynamics becomes dramatically slower after tapping the sample cell several times before rotating it. It is known that tapping has an impact on the packing density and $T_e$ becomes lower. It seems that $T_e$ in the granular system is related with the determination of *L*. Furthermore, we also investigated the gravitational instability of the gelatin system at lower room temperature ($T_r = 19.1^\circ$C), where the polymer network becomes stronger at lower temperature. The time evolution of the gravitational instability in 5–6 wt% at $T_b$ = 40 $^\circ$C and lower $T_r$ is shown as the cross symbol in Fig. 7. It is found that the dynamics becomes much slower although $T_b$ is same. This slowing down of the dynamics is similar to that in the close packing of the granular systems. Those results suggest that the determining factors for *L* is the strength of the network or $T_e$.

After the granular system is fluidized, it is known that a kinematic temperature for the granular systems $T_k$ is important for the granular flow. $T_k$ is high when the grains collide each other and move randomly. Then the granular phase shows the fluid-like behavior for higher $T_k$^27,28^. Here, it is consider that the scaling dynamics for the Rayleigh–Taylor-like instability tends to be held when the grain size is small.

Next we discuss the distribution of the scaled data shown as in Fig. 8. Firstly, statistical distributions in granular experiments are not small. Secondly, the viscosity or the kinematic temperature is not homogeneous in the flowable region. It is different from the fluid system. Finally, a boundary condition between the flowable region and the solid region is different from a rigid wall. From those reasons, a prefactor of the relation between $\lambda$ and *L* may weakly depend on the experimental condition and thus it is considered that the data is deviated from the linear line. Although the data is distributed, we consider that the positive correlation between $\lambda$ and *L* is appropriate as the first approximation. In order to obtain a master curve, microscopic observations and theoretical approach with local properties may be needed.

Then we also discuss the time evolution of $\lambda /\lambda _0$ as shown in Fig. 7c. In fluids, it is known that the relationship given above between $\lambda$ and *L* only applies at the very beginning of the formation of instabilities; the effect of macroscopic flow induced by sedimentation quickly becomes dominant, suppressing the formation of new fingers. Only coarsening is seen at later times. On the other hand, in our gels, we see that $\lambda /\lambda _0$ remains constant for lower $T_b$ for a long time. This leads us to hypothesize that the role of macroscopic flow is different. Figure 5 shows the flow during the time step of the time consecutive images when fingering is seen. It is found the vertical flow is dominant, while the horizontal flow is not observed, or negligible. When fingers which are close together coalesce and the distance between fingers temporarily becomes longer than the most unstable wavelength (see Fig. 4), the Rayleigh–Taylor-like instability can trigger the formation of a new finger. Unlike in fluids, macroscopic flow near the interface in the linear regime is too small to suppress it. New fingers thus arise at intervals of approximately $\lambda$. We emphasize here that $\lambda (t)$ in the linear regime is the most unstable wavelength for *L*(*t*), although $\lambda (t)$ and *L*(*t*) depend on time. It is consistent that the relation of $\lambda \propto L$ in the linear regime remains for a long time. At the later time, flow begins to play a role in the dynamics, making it possible for macroscopic flow to suppress new instabilities; this leads to the deviation of $\lambda /\lambda _0$ from their linear relationship with $L/\lambda _0$. In the granular system, macroscopic air flow is similarly negligible for grains near the interface; the lack of macroscopic flow and air currents can be considered analogous to each other.

Finally, we note that the $\lambda$ is determined by *L*, the density, and the viscosity^36^. In granular system, $\lambda$ is comparable the grain size since the grain is heavy. Meanwhile, $\lambda$ is much larger than the size of the polymer since the viscosity is dominant. Although $\lambda$ is differently determined, the dynamics in both systems becomes similar if it is normalized by $\lambda$. In addition, *L* is crucial for the most unstable wavelength in 3-dimensional fluid systems^36^. Thus, we believe that the analogy between the gel system and the granular system may be established in 3 dimensions, but it should be examined in near future.

## Conclusion

We investigate the connection between granular materials and physical gels. Both systems have a percolated network structure; granular systems have a stress network while physical gels have a polymer chain network. The Rayleigh–Taylor-like instability is induced by the disconnection of the network near the interface. We identify analogous dynamics in both systems: (i) a coexistence between fluidized and non-flowing regions; (ii) small wavelength instability for a long time; (iii) the emergence of new fingers at later stages. It is found that the dynamical behavior can be scaled by the thickness of the flowable region *L*; this means that the dominant parameter governing the instability dynamics is *L* in both systems. It is also suggested that the time evolution of *L* is determined by the strength of the network in the granular region or the upper gel region. Thus, we conclude that the Rayleigh–Taylor-like instability in the granular system is also governed by the height of the fluidized layer over time. Though this work focuses on analogies as applied to the Rayleigh–Taylor-like instability, our method may be useful for investigation of other instability phenomena in granular systems. This may lead to a deeper understanding of granular media, with an impact anticipated for industrial applications and measures to prevent certain types of natural disasters.

## Materials and experimental methods

### Gelatin solution systems

Our experimental setup consists of a glass sample chamber attached to a heating stage (Fig. 1a). The sample chamber is a quasi two-dimensional Hele-Shaw cells for facile analysis of the flow. To analyze the distance between the fingers quantitatively, the cell size is ($H_c$, $L_c$, $W_c$) = (30 mm, 156 mm, 2.4 mm). $L_c$ is large for the gel systems in order to analyze $\lambda$ quantitatively. Note that thermal convection does not occur during the Rayleigh–Taylor-like instability at $H_c$ = 30 mm^37,38^. The thickness of the lower layer was set to 5 mm; the upper layer was set to 25 mm. Here, we used gelatin purchased from Wako Pure Chemical Industries Co., Ltd., and made gelatin solutions (3 wt%, 4 wt%, 5 wt%, 6 wt%, 8 wt%, 10 wt%, 12 wt%, and 14 wt% concentration) using pure water as a solvent. We add polystyrene latex (size: $10.3\mu \hbox {m}$, density: 1.05 g/$\hbox {cm}^3$) to the upper layer (higher concentration) as tracer particles for visualization of the velocity field and the concentration of polystyrene latex is 0.05 wt%. The density of the latex is close to the gelatin solution, thus the sedimentation of the latex is quite slower than the dynamics of the Rayleigh–Taylor-like instability. In addition, we examined the flow when we add a dye (rhodamine 6G) in the upper layer instead of the latex particles and then it is found that the flow with the dye is quantitatively same as that with the latex. Thus, we conclude that the latex has no effect for the Rayleigh–Taylor-like instability. Images were recorded with a digital camera (HC-V520M, Panasonic Co.) at 1 s intervals. Details of the experimental method can also be found in Ref.^23^. The room temperature $T_r$ was set to 21.3 °C with a standard air conditioner. Since $T_r$ is below the sol-gel transition temperature, the gelatin solution remains the gel state before the heating. We controlled the temperature of the bottom surface $T_b$ using a temperature controller (S100 Blast Co.). The upper and lower liquids are gelled gelatin solutions; we can change the density and viscosity by changing the concentration of the gelatin solution. Firstly, we inject the gelatin solution with higher concentration and cool it until it solidifies. The gelatin solution with lower concentration is layered over the solid layer with a higher concentration of gelatin. After this is also gelled, the entire experimental cell is rotated, yielding a static initial interface with a heavier layer on top.

The sol-gel transition temperature was evaluated by employing a tube inversion method^39^. The gelatin solution is in a gel state under the solid line in Fig. 2a; it is in a sol (fluid) state above the solid line. It is known that a gelatin solution is a Newtonian fluid above the sol-gel transition temperature $T_s$ up to a high shear rate ($\sim$ 1000 $\hbox {s}^{-1}$)^40^. Thus, gelatin in the fluidized region can be treated as a Newtonian fluid in this experiment.

### Granular systems

In order to observe gravitational instability in granular materials, we purchased grains from Kitanihon Sangyo Co. with size $0.425 \pm 0.150$ mm. The density of the grain is 2.62 g/$\hbox {cm}^3$. The packing fraction is calculated from the mass and the volume of the grain layer. The instability pattern of the granular systems is recorded using a high-speed digital camera (VW-600M, KEYENCE Co.) with a frame rate of 500 fps. The cell size is ($H_c$, $L_c$, $W_c$) =(150 mm, 75 mm, 1.2 mm) and (90 mm, 65 mm, 2.4 mm) for the granular systems. There are artificial disturbances when the granular system is rotated manually to initiate the instability phenomenon. According to Ref.^17^, the effects of those disturbances remain in short time. Thus $H_c$ should be large. Meanwhile, those disturbances become larger with increasing the weight of the system. Thus, $L_c$ in the granular system is shorter than that in the gel system.
